# EBCR: Empirical Bayes concordance ratio method to improve similarity measurement in memory-based collaborative filtering

**DOI:** 10.1371/journal.pone.0255929

**Published:** 2021-08-09

**Authors:** Yu Du, Nicolas Sutton-Charani, Sylvie Ranwez, Vincent Ranwez

**Affiliations:** 1 EuroMov Digital Health in Motion, Univ Montpellier, IMT Mines Ales, Ales, France; 2 UMR AGAP Institut, Univ Montpellier, CIRAD, INRAE, Institut Agro, Montpellier, France; Lingnan University, HONG KONG

## Abstract

Recommender systems aim to provide users with a selection of items, based on predicting their preferences for items they have not yet rated, thus helping them filter out irrelevant ones from a large product catalogue. Collaborative filtering is a widely used mechanism to predict a particular user’s interest in a given item, based on feedback from neighbour users with similar tastes. The way the user’s neighbourhood is identified has a significant impact on prediction accuracy. Most methods estimate user proximity from ratings they assigned to co-rated items, regardless of their number. This paper introduces a similarity adjustment taking into account the number of co-ratings. The proposed method is based on a concordance ratio representing the probability that two users share the same taste for a new item. The probabilities are further adjusted by using the Empirical Bayes inference method before being used to weight similarities. The proposed approach improves existing similarity measures without increasing time complexity and the adjustment can be combined with all existing similarity measures. Experiments conducted on benchmark datasets confirmed that the proposed method systematically improved the recommender system’s prediction accuracy performance for all considered similarity measures.

## Introduction

In recent years, the exponential increase in available data and information overload have led to a growing call for recommender systems. Many e-commerce sites rely on them to help users efficiently navigate through ever-increasing numbers and sizes of product catalogues. As the offer increases, effective recommenders become essential to guide users through the plethora of offers available. Recommender systems are often classified in three categories: content-based filtering, that recommends items whose contents are similar to the user’s previously liked items [1]; collaborative filtering (*CF*), that targets items appreciated by users with similar tastes [2] and hybrid recommenders combining the two previously described approaches [3].

Compared to the content-based approach, that relies mainly on item attributes, the collaborative filtering technique takes advantage of taste similarities, which generally leads to more accurate user preference predictions [4]. Starting from an incomplete (typically sparse) *user-item* ratings matrix, collaborative filtering aims at filling the matrix by predicting user ratings for items that they have not yet evaluated. To this end, two types of collaborative filtering (*CF*) approaches have been proposed: memory-based *CF* and model-based *CF*. Memory-based approaches (also called neighbourhood-based approaches) infer a particular user’s missing rating by averaging the rating values of the *k* nearest neighbours (KNN) having rated the item. This neighbourhood is directly computed using the ratings matrix. In contrast, model-based approaches first compress the matrix in a reduced-dimension latent space that will serve as a basis for the predictive variables of supervized learning models [5]. For instance, the matrix factorization technique can be used to obtain a reduced-dimension ratings matrix [6].

Some recommender systems proceed in two steps, first estimating user ratings (rating prediction) and then recommending the list of the top rated items (top-N recommendations) while others skip the first phase and directly produce a list of recommended items, hence making top-N recommendations without rating predictions. Although returning the list of suggested items may be considered as the real goal of recommender systems, with item ratings regarded as just an intermediate step, obtaining reliable rating estimations may have its own interest. For instance it may detect that none of the items really fit the user’s needs (the top items having low rating estimations); it could prioritize the suggested items; or combine users ratings to obtain a list of suggested items for a specific group of users, e.g. for social recommendations or customer segmentation [7]. Though related, the rating prediction task and the top-N recommendation task are fundamentally different, one being somehow a regression task while the other is a classification one. The current trend towards deep learning approaches has strongly impacted recent recommender system developments [8, 9]. The development of deep learning approaches has mostly focused on the top-N recommendation task, at which they now excel. Nevertheless, as pointed out in [10], most published recommender systems using deep neural networks do not outperform cutting-edge memory-based approaches, especially for rating prediction. Indeed, as emphasized in [11, 12], the methods that perform best on top-N recommendations are not those that perform best on rating prediction. For the top-N recommendation task, the difference between disliked and abhorred items does not matter, while it is crucial for the rating prediction task. This paper focuses on improving rating predictions, a task for which, as confirmed by the conducted experiments, deep learning approaches are not always the best solutions. We believe that improving rating predictions could benefit many applications, such as suggesting a restaurant to a group of friends, since someone who does not mind going to a pizzeria is drastically different from someone who absolutely refuses to (e.g. because of a gluten allergy).

The aim of the paper is to improve rating predictions by refining the similarity estimations involved in computing users’ neighbourhoods in the memory-based collaborative filtering context. To measure the similarity between two users, most standard metrics are restricted to their co-rated items. This limitation makes similarity estimations quite unreliable for users who have few rated items in common. In addition, items may be evaluated differently from others based on the user’s background knowledge or personal character. Therefore, it is also necessary to consider the disparity of rating distributions between users [13].

We propose an approach, namely EBCR (Empirical Bayes Concordance Ratio), for refining the estimation of pairwise user similarities by taking into account: i) the disparity of rating distributions between users (the CR part), and ii) the number of co-rated items (the EB part). The proposed similarity adjustment is based on a weighting concordance ratio smoothed by using the Empirical Bayes model. Its evaluation on benchmark datasets shows encouraging predictive improvements.

## State of the art

### Memory-based collaborative filtering

A recommender system is designed to predict, for each user *u* and each item *i* that he/she has not yet rated, the rating ${\hat{r}}_{u,i}$ that *u* would assign to *i*. Two memory-based collaborative filtering approaches have been proposed: the user-based and the item-based approach. To determine the value of ${\hat{r}}_{u,i}$, the former approach makes use of ratings assigned to item *i* by users with tastes similar to user *u*’s, whereas the latter approach takes advantage of user *u*’s own ratings on items similar to item *i* [14]. Finally the items with the highest predicted ratings are recommended to user *u*. Below, the formalization of the proposed work is based on the user-based collaborative filtering context. Nevertheless, it is worth noticing that the proposed work can be directly transposed to any item-based one. The item-based approach is particularly efficient in cases where the number of users is much larger than the number of items, thus requiring much less computer memory to build the item similarity matrix.

To predict ${\hat{r}}_{u,i}$, the memory-based collaborative filtering technique proceeds in two consecutive steps: 1) neighbourhood identification and 2) rating prediction. We briefly summarize these two steps in the following sections.

#### Neighbourhood identification

During the first phase, the algorithm aims to determine a neighbourhood for the target user *u*. To this end, the *k* nearest neighbours (KNN) approach, which is based on pairwise similarities between users, is typically applied. Let *U* be the sets of users and *I* the set of items. Then, let *R* be the |*U*| × |*I*| incomplete ratings matrix and *r*_*u*,*i*_ be the rating given to item *i* by user *u*. The neighbourhood identification relies on the construction of a |*U*| × |*U*| similarity matrix *S* (based on the ratings matrix *R*), where *S*_*u*,*v*_ denotes the similarity between users *u* and *v*. As each user’s taste is represented by a rating vector i.e. **u** = (*r*_*u*,*i*_, *i* = 1, 2, …, |*I*|), the similarity between users *u* and *v* can be estimated by the proximity between their two rating vectors **u** and **v**.

The cosine similarity is a metric initially used in the information retrieval field to measure the similarity between documents represented by word frequency vectors [15]. It is one of the most commonly used similarities in the memory-based collaborative filtering context. Formally, the cosine similarity between users *u* and *v*, denoted by *sim*_*COS*_(*u*, *v*), is measured by the cosine of *u* and *v*’s rating vectors, restricted to their co-rated items *I*_*u*,*v*_ = {*i* ∈ *I*|*r*_*u*,*i*_ ≠ ∅ and *r*_*v*,*i*_ ≠ ∅}.

Instead of directly measuring the similarity between two vectors, as with *sim*_*COS*_, other metrics first calculate a distance between these vectors and then convert it into a similarity [13]. To calculate the distance between *u* and *v*, they can be considered as two points positioned in an Euclidean space of dimension |*I*_*u*,*v*_| where the distance between vectors **u** and **v** can easily be computed. The simple Euclidean distance tends to be biased, in the sense that users having rated many items in common will, by construction, appear to be more distant than those for whom there are only a few co-ratings. It is therefore preferable to use the Mean Squared Distance (MSD) whose definition, recalled in Eq (1), is inspired by the square error of a statistical estimator [16]. Several generic strategies may be applied to convert a distance *dist* into a similarity *sim*, such as calculating its inverse, i.e. *dist*^−1^ or using the 1 − *dist* formula once the distance is normalized into [0, 1]. Eq (2) shows an example of the conversion of Eq (1) to similarity. Note that 1 is added to the denominator to avoid a possible division by 0.

$$\begin{array}{c} MSD\left(u,v\right)=\frac{\sum_{i\in I_{u,v}}{\left(r_{u,i}-r_{v,i}\right)}^{2}}{|I_{u,v}|} \end{array}$$ (1)

$$\begin{array}{c} sim_{MSD}\left(u,v\right)=\frac{1}{MSD(u,v)+1} \end{array}$$ (2)

Another widely used similarity metric [17] in the collaborative filtering context is the Pearson correlation coefficient (PCC), i.e. *sim*_*PCC*_(*u*, *v*), which measures the linear correlation of two vectors. Note that in contrast to *sim*_*COS*_ and *sim*_*MSD*_ whose range is [0, 1], the range of *sim*_*PCC*_ is [−1, 1]. This difference could impact the evaluation behaviour of the similarity adjustment procedures. Therefore, in this article, *sim*_*PCC*_(*u*, *v*) is normalized to obtain values in the [0, 1] interval using Eq (3):

$$\begin{array}{c} sim_{NormPCC}\left(u,v\right)=\frac{sim_{PCC}\left(u,v\right)+1}{2} \end{array}$$ (3)

The resulting similarity measure is denoted by *NormPCC*, which stands for the normalized Pearson correlation coefficient.

#### Rating prediction

After having determined the neighbourhood for user *u*, during the rating prediction phase, the memory-based collaborative filtering algorithm makes use of the available ratings within the previously determined neighbourhood. The rating task is typically achieved by weighted averaging *u*’s neighbours’ ratings, in which neighbour weights are based on their similarity values with respect to the target user *u*, so that closer neighbours have more impacts. Eq (4) illustrates the weighted average method for the prediction of ${\hat{r}}_{u,i}$. The *sim*(*u*, *v*) term in the equation represents the similarity value between user *u* and *v* (one of *u*’s neighbours). The *N*_*u*_ term represents the determined neighbourhood for user *u*.

$$\begin{array}{c} {\hat{r}}_{u,i}=\frac{\sum_{v\in N_{u}}r_{v,i}*sim\left(u,v\right)}{\sum_{v\in N_{u}}sim\left(u,v\right)} \end{array}$$ (4)

Generally, the way users rate items is influenced by their personality, mood and context. Thus, on a 1 to 5 rating scale, an optimistic (resp. pessimistic) user will seldom give a score of 1 (resp. 5) to an item even if he/she does not like it (resp. likes it). Users’ rating distributions can therefore be shifted or compressed relative to each other. Rating normalization is often used to overcome this problem [13, 18]. The z-score normalization, i.e. Eq (5) is an adaptation of Eq (4), in which the ratings of each user are centred and reduced:

$$\begin{array}{c} {\hat{r}}_{u,i}={\bar{r}}_{u}+\sigma_{u}\frac{\sum_{v\in N_{u}}\left[\left(\frac{r_{v,i}-{\bar{r}}_{v}}{\sigma_{v}}\right)*sim\left(u,v\right)\right]}{\sum_{v\in N_{u}}sim\left(u,v\right)} \end{array}$$ (5)

with ${\bar{r}}_{u}$ and *σ*_*u*_ (resp. ${\bar{r}}_{v}$ and *σ*_*v*_) representing the average and the standard deviation of *u*’s (resp. *v*’s) ratings.

The contribution of the proposed approach is to adjust the similarity measurement in the first phase of the memory-based collaborative filtering algorithm in order to identify a more reliable neighbourhood to be used for the rating prediction phase. In the following section, we discuss the disadvantages of the conventional similarity measures and present some existing works dealing with these drawbacks.

### Disadvantages of conventional similarity measures

Although widely used in the memory-based collaborative filtering context, the similarity measures described in the previous section have disadvantages [19]. The Pearson correlation approach first positions each observation, i.e. (*r*_*u*,*i*_, *r*_*v*,*i*_) ∀*i* ∈ *I*_*u*,*v*_, in a 2-dimensional space and then calculates a linear correlation. However, this approach is unreliable for small *I*_*u*,*v*_ sets. Consider the following example: **u** = (1, 3, 2, ∅, 1), **v** = (1, ∅, ∅, 5, ∅) and **w** = (1, 2, 2, ∅, 1), i.e. three rating vectors that represent the preferences of users *u*, *v* and *w* for the same five items. With the Pearson correlation approach, the obtained similarities are: *sim*(*u*, *v*) = 1 > *sim*(*u*, *w*) = 0.905, which is not desirable because the assumed similarity between *u* and *v* is based on a single observation, while the similarity between *u* and *w* is based on four observations. It is thus more sure that *u* and *w* have similar tastes than *u* and *v*. It seems therefore more relevant to use *w*’s ratings rather than *v*’s to predict what rating *u* would give to the fourth item as the prediction would be more reliable. The same phenomenon can be observed with *sim*_*COS*_, since *sim*_*COS*_(*u*, *v*) = 1 > *sim*_*COS*_(*u*, *w*) = 0.9798. As mentioned previously, the main shortcoming of these similarity metrics is that they only consider rating distributions restricted to co-rated items. In other words, they overlook the number of items co-evaluated by users and thus the fact that the reliability of the user similarity prediction increases with the number of co-rated items. Managing the resulting uncertainty could make the neighbourhood computations smoother and the predictions more accurate.

It would be interesting to attempt to remedy this drawback by discounting similarities estimated from only a few co-rated items. In [13], the authors proposed the *significance weighting* factor (see Eq (6)), which penalizes user similarities when the number of co-rated items is below a threshold *t*. They showed that a *CF*-based recommender system using *significance weighting* performs better in terms of rating accuracy. The disadvantage of this approach is its potential lack of genericity. As the authors only evaluated it with the Pearson correlation coefficient measure on a single dataset, it is unknown how it would perform with other similarity measures. Moreover, how to fix the threshold *t* is not straightforward. The authors advise fixing it to 50 after having tried various *t* values. Here again, the genericity may be questioned. In addition, the *significance weighting* approach does not consider rating distributions of other users. As the *significance weighting* method, which can be combined with any similarity measure, the approach proposed in this paper further uses the distribution of all estimated similarities in order to improve their predictive abilities.

$$\begin{array}{c} sim_{t}\left(u,v\right)=\{\begin{array}{cc} sim\left(u,v\right)\times \frac{|I_{u,v}|}{t}, & \text{if}\;|I_{u,v}|<t,\\ sim(u,v), & \text{otherwise.} \end{array} \end{array}$$ (6)

## Contribution in terms of similarity measurement

In this section, the details of the proposed approach are presented. The notion of the proposed Concordance Ratio (the CR part) is detailed, followed by the description of the proposed adjustment method performed by the Empirical Bayes model (the EB part).

### Relaxation of similarity by a concordance ratio

In the collaborative filtering context, two users are generally considered similar when there is concordance between their taste profiles, i.e. their ratings. Here, we propose firstly to discretize user ratings into three ordered categories i.e. *like*, *neutral* and *dislike* reflecting the user’s taste for an item, as defined by Definition 1. To alleviate the disparity of rating behaviours among users, we carry out this discretization based on the z-score as detailed in Eq (7). The parameter *a* ∈ [0, 1] used in Eq (7) is a hyper-parameter, which parameterizes the size of the *neutral* area.

**Definition 1**. (**Discretization of user tastes**). *Let a* ∈ [0, 1], *the taste of user u on the item i*, *discretized by the operator T, is defined as follows*:

$$\begin{array}{c} T\left(r_{u,i}\right)=\{\begin{array}{cc} \text{like}, & \text{if}\;\frac{r_{u,i}-{\bar{r}}_{u}}{\sigma_{u}}>a,\\ \text{dislike}, & \text{if}\;\frac{r_{u,i}-{\bar{r}}_{u}}{\sigma_{u}}<-a,\\ \text{neutral}, & \text{otherwise}. \end{array} \end{array}$$ (7)

Based on this discretization, the concept of *concordance* between user tastes is defined by Definition 2. The proposed *concordance* term is related to two users’ ratings of a single item and we use the term “concordant ratio” for the measure of the overall profile concordance of two users, as defined by Definition 3.

**Definition 2**. (**Concordance of tastes**). *Let u* ∈ *U and v* ∈ *U be two users and i* ∈ *I*_*u*,*v*_ *be one of their co-rated items*. *The pair of ratings* (*r*_*u*,*i*_, *r*_*v*,*i*_) *is defined as concordant, if and only if T*(*r*_*u*,*i*_) = *T*(*r*_*v*,*i*_). *The set of items with a concordant rating for u and v is denoted C*_*u*,*v*_ = {*i* ∈ *I*_*u*,*v*_ | *T*(*r*_*u*,*i*_) = *T*(*r*_*v*,*i*_)}.

The definition of *concordance* is based on the approach of [20], in which the authors used the *concordant* notion with respect to privacy problems in recommender systems.

**Definition 3**. (**Concordance ratio**). *Considering users u* ∈ *U and v* ∈ *U*, *the concordance ratio is defined as* $CR\left(u,v\right)=\frac{|C_{u,v}|}{|I_{u,v}|}$, *which corresponds to the proportion of items co-rated in a concordant manner by u and v among all of their co-rated items*.

The concordance ratio for users *u* and *v* can be interpreted as the probability that *u* and *v* would have the same taste for a new item. We propose new similarity measures denoted as *sim*_*CR*_ and defined as the product of the concordance ratio with one of the previously presented similarity metrics i.e. *sim*_*COS*_(*u*, *v*), *sim*_*MSD*_(*u*, *v*) and *sim*_*PCC*_(*u*, *v*). The metric *sim*_*CR*_(*u*, *v*), i.e. Eq (8), corresponds to a discounting of *sim*(*u*, *v*) with respect to the taste concordance between users *u* and *v*.

$$\begin{array}{c} \begin{array}{cc} sim_{CR}\left(u,v\right) & =CR\left(u,v\right)\times sim_{}\left(u,v\right) \end{array} \end{array}$$ (8)

### Adjustment of the concordance ratio via the Empirical Bayes model

The proposed concordance ratio alleviates the problem related to the disparity of rating distributions between users, as the discretization of user tastes is carried out in the same way for each user by considering their specific rating distribution. For example, considering a 1 to 5 rating scale, a rating of 3 for the same item could express a different taste (e.g. like, dislike, neutral) between optimistic and pessimistic users. Nevertheless, this manner of measuring the concordance between users is still not fully reliable. For example, considering the following two concordance ratios:

$$CR\left(u,v\right)=\frac{|C_{u,v}|}{|I_{u,v}|}=\frac{3}{3}=1\;\text{and}\;CR\left(u,w\right)=\frac{|C_{u,w}|}{|I_{u,w}|}=\frac{290}{300}=0.97.$$

Users *u* and *v* seem to have more similar tastes than *u* and *w* when considering their concordance ratio values. However, *u* and *v* only shared their opinions on three items, any similarity estimation between them is necessarily highly uncertain and should be smoothed, as they could have a completely different opinion on a fourth item. To predict *u*’s ratings, it seems therefore preferable to rely on *w*’s ratings rather than on *v*’s. Even if *CR*(*u*, *w*) is a little lower than *CR*(*u*, *v*), its estimation is much more reliable since it is based on hundreds of evaluations. This example highlights the need for an adjustment to the concordance ratios, that should take into account the number of co-rated items between users in order to adjust the smoothing range according to some information criteria.

Laplace or *additive* smoothing is widely used in statistics and machine learning domains in order to smooth multinomial probability estimations with regard to the size of the considered sample. In the context of concordance ratio adjustment, it is defined by Eq (9) where *α* is the *pseudocount* parameter:

$$\begin{array}{c} Laplace\left(u,v\right)=\frac{|C_{u,v}|+\alpha }{|I_{u,v}|+2\alpha } \end{array}$$ (9)

From a Bayesian point of view, it corresponds to the update of a beta probability given a non-informative (i.e. uniform) prior distribution. In this article we propose to make use of the information contained in the whole sample to determine the parameters to be used to adjust (or smooth) the proposed concordance ratios, rather than using predefined *α* and 2*α* values to do so.

In [21], the authors proposed to apply the Empirical Bayes model to correct the probability of a successful shot for baseball players who had not played much during a season. Inspired by this work, we propose to penalize the concordance ratios based on few co-rated items (i.e. small |*I*_*u*,*v*_| sets). The approach considers that the proposed concordance ratio ($\frac{|C_{u,v}|}{|I_{u,v}|}$) is analogous to the probability of a successful shot, i.e. $\frac{\text{number}\;\text{of}\;\text{successful}\;\text{shots}}{\text{total}\;\text{number}\;\text{of}\;\text{shots}}$ in the baseball case. Specifically, we assume that the concordance ratios observed on the total sample follow a beta distribution of *Beta*(*α*_0_, *β*_0_), which could be used as a prior distribution. *α*_0_ and *β*_0_ are the two hyper-parameters of the beta distribution, which determines its shape. The values of *α*_0_ and *β*_0_ are estimated by maximizing the likelihood of these parameters given the proportions observed over the entire dataset. Afterwards, each concordance ratio value is replaced, based on this *Beta* distribution, by the corresponding *posterior* distribution which shifts it more or less towards the average value of all the observed proportions, i.e. the expected observed distribution value. Hence, a ratio based on few values, e.g. $\frac{3}{3}$, is highly corrected to become closer to the *Beta* distribution’s expectation, i.e. $\frac{\alpha_{0}}{\alpha_{0}+\beta_{0}}$, while a ratio estimated on many values e.g. $\frac{290}{300}$, will remain almost unchanged. This approach allows the smoothed concordance ratio to take the number of co-rated items (|*I*_*u*,*v*_|) into account. Moreover, this smoothing takes advantage of information about other users’ tastes, which could be considered as a collaborative extra step. Thus, we define the Empirical Bayes Concordance Ratio (EBCR) as the smoothed or adjusted version of *CR*(*u*, *v*) according to Empirical Bayes smoothing:

$$\begin{array}{c} EBCR\left(u,v\right)=\frac{|C_{u,v}|+\alpha_{0}}{|I_{u,v}|+\alpha_{0}+\beta_{0}} \end{array}$$ (10)

Finally, any similarity measure could be adjusted by multiplying it by the EBCR weighting term. For example, the term *EBCR_COS* denotes the adjusted measure for the *COS* (Cosine) similarity, as illustrated by Eq (11).

$$\begin{array}{c} sim_{EBCR\_COS}\left(u,v\right)=EBCR\left(u,v\right)\times sim_{COS}\left(u,v\right) \end{array}$$ (11)

The adjustment of *α*_0_ and *β*_0_ is a pre-processing step that is performed only once. Note that |*I*_*u*,*v*_| must be identified to be able to calculate *sim*_*COS*_(*u*, *v*), |*C*_*u*,*v*_| is calculated in a time complexity of *O*(|*I*_*u*,*v*_|), as is also the case for *sim*_*COS*_(*u*, *v*). Therefore, computing the similarity between *u* and *v* has the same complexity in terms of computation time regardless of whether or not it integrates the EBCR weighting.

## Assessment

### Compared approaches

This section briefly introduces the compared approaches. First, we would like to assess the impact of integrating similarity metrics with the proposed EBCR adjustment during the rating prediction phase within memory-based collaborative filtering. Thus, we compare the memory-based collaborative filtering method using basic similarity metrics with the one using the corresponding adjusted similarity metrics. Second, we compare the proposed EBCR method with other previously presented similarity adjustment methods, i.e. the *significance weighting* approach (Eq (6)) and the *Laplace* smoothing approach (Eq (9)). Third, we are also interested in broadening the assessment by comparing the prediction performances of the proposed memory-based CF approach with the performances of model-based approaches. The latter mainly leverage dimensionality reduction techniques, such as matrix factorization and have proven to be highly accurate and flexible, notably when the ratings matrix is sparse [22]. To this end, we opted to complement the assessment experiments by considering the following models: the *Baseline* [22], SVD (Singular Value Decomposition) [6], SVD++ [23] models and the NeuMF (Neural Matrix Factorization) [24] model, which is based on deep neural networks. Before presenting the assessment of our approach, we briefly describe each of these models.

#### Baseline

The *Baseline* model is considered as one of the reference approaches for model-based collaborative filtering. A priori, the rating of an item *i* given by a user *u* depends on the item and the user. An item may be more or less appreciated by the public and a user may have a more or less severe rating behaviour. Ratings can therefore be estimated using a simple linear model with two explanatory variables (the user and the item). Let *μ* be the overall average of all ratings in the dataset, then the *Baseline* prediction of ${\hat{r}}_{u,i}$ is defined by Eq (12) as:

$$\begin{array}{c} \begin{array}{c} {\hat{r}}_{u,i}=\mu +b_{u}+b_{i} \end{array} \end{array}$$ (12)

where *b*_*u*_ and *b*_*i*_ represent respectively the separate user and item effects. Readers may refer to [22] for further details on the *Baseline* approach and its variants.

#### SVD and SVD++

The SVD (singular value decomposition) approach is one of the most popular model-based CF approaches. The model applies matrix factorization techniques to map users and items to a joint latent factor space of reduced dimensionality *d* and the learnt representations are then used to predict ratings. Formally, each item *i* is associated with a factor vector $q_{i}\in {I\;R}^{d}$ and each user *u* is associated with a factor vector $p_{u}\in {I\;R}^{d}$. The inner product of these two vectors in the latent vector space represents the predicted rating, i.e. ${\hat{r}}_{u,i}=q_{i}^{T}p_{u}$. The learning of these factor vectors is typically conducted by optimizing the regularized loss function on the training set (i.e. Eq (13)) using stochastic gradient descent.

$$\begin{array}{c} \min_{p,q}\sum_{r_{u,i}\in R_{train}}{\left(r_{u,i}-q_{i}^{T}p_{u}\right)}^{2}+\lambda (∥q_{i}{∥}^{2}+∥p_{u}{∥}^{2}) \end{array}$$ (13)

The SVD++ approach is an extension of the SVD approach which considers the perspective of implicit feedback from users.

#### Neural Matrix Factorization

NeuMF is a collaborative filtering model based on deep neural networks. In general, recommender systems based on neural networks take the user ratings matrix as the input layer and generate a score for each user-item pair on the output layer. The hidden layers, i.e. a multi-layer neural architecture, reveal the latent structures of user–item interactions. The NeuMF approach [24] is one of the most cited deep neural network-based recommendation approaches [10]. Specifically, the approach concatenates the last hidden layers of two neural networks: the GMF (Generalized Matrix Factorization) and the MLP (Multi-Layer Perceptron). The main idea of the GMF neural architecture is to generalize the matrix factorization model i.e. Eq (13), in which latent factors are treated equally and a linear function (the inner product) is used to model the user-item interaction. The GMF neural network is capable of learning different weights for each latent factor. In addition, the use of a non-linear activation function at the output layer allows the network to consider non-linear user-item interactions. The MLP neural network, unlike the GMF one which uses only a fixed element-wise product of **p**_*u*_ and **q**_*i*_, enables the learning of more subtle user-item interactions by adding multiple hidden layers on the input layer, consisting of the concatenated vector of **p**_*u*_ and **q**_*i*_. Finally, the last hidden layers of GMF and MLP are combined to generate the final output score of a given user-item pair.

The following subsections describe the datasets and evaluation metrics used, before detailing the experimental protocol.

### Datasets

The experimental assessment of the proposed method is based on three benchmark datasets: 1) the *MovieLens-100K* dataset, which consists of 100,000 ratings from 943 users on 1,682 movies; 2) the *MovieLens-1M* dataset, which represents a collection of 1,000,209 ratings assigned by 6,040 users to 3,900 movies and 3) the *Jester* dataset, which contains over 1.7 million ratings of 140 jokes from 59,132 anonymous users. Collected by the GroupLens Research Project at the University of Minnesota, the ratings in the two movie datasets are based on a 5-point scale [25]. The *Jester* joke dataset, in which the ratings are specified on a -10 to 10 continuous scale, was derived from the Jester joke recommender system [26]. These three datasets are well-known benchmark datasets in the recommender system domain [27]. Characteristic details of the above datasets are given in Table 1.

**Table 1:** Dataset characteristics.

dataset	#users	#items	#ratings	rating scale	density	domain
MovieLens-100K	943	1682	100K	[1, 5]	6.30%	Movie
MovieLens-1M	6,040	3,900	1M	[1, 5]	4.47%	Movie
Jester	59.1K	140	1.7M	[-10, 10]	20.53%	Joke

The dataset density, i.e. the inverse of the sparsity, represents the percentage of cells in the full user-item matrix that contain rating values.

### Evaluation metrics

In the literature, the accuracy assessment of recommender systems is typically divided into two classes of evaluation scenarios: i) *rating prediction* and ii) *top-N* recommendations. Evaluation metrics involved in i), e.g. RMSE (Root-Mean-Square Error), measure how well a recommender system would perform in the task of predicting user ratings, which is typically done by measuring the differences between the predicted ratings and the actual ones for those items whose rating values are known. On the other hand, evaluation metrics based on the *top-N* recommendations, e.g. Precision, Recall, NDCG (Normalized Discounted Cumulative Gain) etc. aim to evaluate a list of recommendations as a whole. This kind of evaluation scenario measures how well the recommender system would perform at ranking the relevant items for the user e.g. the ones that he/she has liked or has interacted with, ahead of their unrated items.

The *rating prediction* based evaluation approach is widely adopted in the context of recommender systems, notably for comparing collaborative filtering algorithms, e.g. the Netflix Prize [28]. Although effective, researchers have also claimed that *rating prediction* based metrics could be inaccurate in some cases. For that reason, researchers have investigated the comparison and relationship between these two classes of evaluation metrics [11, 12, 29]. In [11], the authors found that there is little/no correlation between these two kinds of metrics. In other words, an algorithm that performs well at predicting user ratings does not necessarily perform equally well when dealing with the top-N recommendations task. In [12], the authors compared various algorithms in a top-N recommendations context and found that algorithms that are designed to rank items for top-N recommendations could outperform algorithms that are good at predicting ratings.

The current work deals with the collaborative filtering recommendation scenario, which is typically evaluated by *rating prediction* based metrics. Thus, we adopted two basic rating prediction based metrics, i.e. the mean-absolute-error (MAE) and the root-mean-square-error (RMSE):

$$\begin{array}{c} MAE=\frac{\sum_{u,i}\left|r_{u,i}-{\hat{r}}_{u,i}\right|}{\text{NP}} \end{array}$$ (14)

$$\begin{array}{c} RMSE=\sqrt{\frac{\sum_{u,i}{\left(r_{u,i}-{\hat{r}}_{u,i}\right)}^{2}}{\text{NP}}} \end{array}$$ (15)

where *NP* denotes the number of predicted ratings, i.e. the size of the test set, while *r*_*u*,*i*_ and ${\hat{r}}_{u,i}$ respectively denote the actual and predicted ratings. Lower MAE and RMSE values correspond to more accurate rating predictions.

### Experimental protocol

This section details the experimental protocol used to evaluate the proposed EBCR method.

#### Three different types of method comparisons

The proposed EBCR adjustment is assessed with respect to three types of comparisons:

1. Impact of the proposed EBCR adjustment for different similarity measures during the memory-based collaborative filtering prediction process. To this end, we compare collaborative filtering approaches using the basic similarity measures, i.e. *sim*_*COS*_, *sim*_*MSD*_ and *sim*_*NormPCC*_ with the ones integrating the EBCR adjustment. We adopt names of the similarity measures to denote the basic approaches (e.g. *COS_KNN* for the approach with cosine similarity). Note that the “*KNN*” (k-nearest neighbours) term is combined with the similarity label, as different neighbourhood sizes are considered for evaluation. Respectively, the “EBCR” term is added to denote the EBCR adjustment, e.g. *EBCR_COS_KNN*. This type of comparison allows to evaluate the relevance and the genericity of the proposed adjustment.
2. Comparison with other state-of-the-art similarity adjustment approaches. To this end, we compare the proposed EBCR method with two existing adjustments: the *significance weighting* factor, i.e. Eq (6) and the Laplace smoothing, i.e. Eq (9). The former is denoted by the *SW* term (e.g. *SW_COS_KNN*) while the latter is denoted by the *LS* one (e.g. *LS_COS_KNN*).
3. Comparison with state-of-the-art model-based collaborative filtering approaches. The considered models are the following: *Baseline*, SVD, SVD++ and NeuMF. Details of these models are provided at the beginning of the Assessment section. For this type of comparison, we denote the proposed approach as “EBCR” for short.

#### Dataset split

For the experiments, we performed a 5-fold cross-validation. For each of the three considered datasets, we randomly separated the entire dataset into 5 sub-samples of the same size and selected one for validation (test set), while the four others were used as the training set. We then repeated this process by selecting another sample as the test set, etc. hence resulting in 5 different MAE and RMSE values. All of the compared approaches were trained on the same training sets and evaluated on the same test sets. The average of the 5 MAE and 5 RMSE values were finally considered for each compared model.

#### Chosen parameters for models

**Fig 1:**
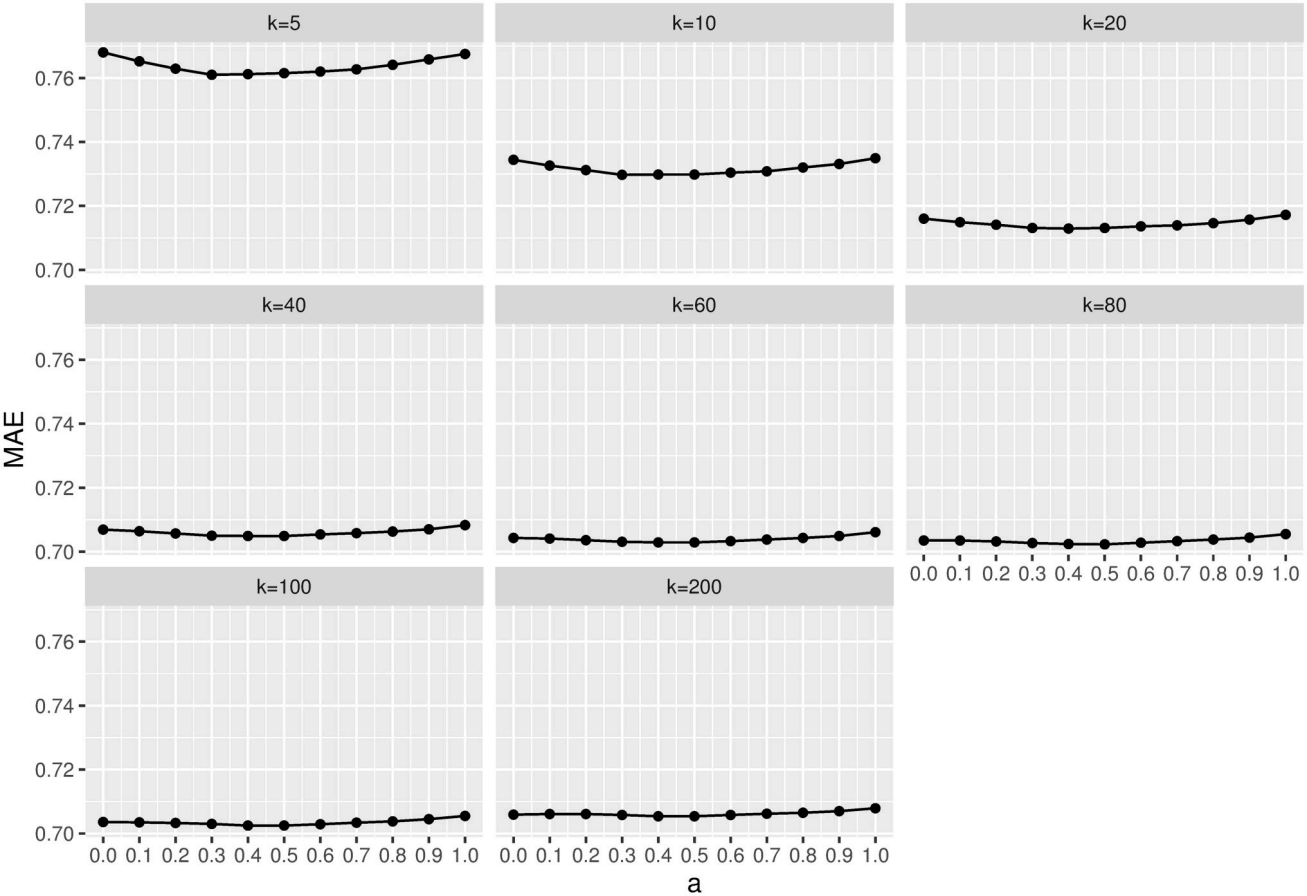
Experimental results of EBCR with different *a* values (cf. Eq (7)) for different neighbourhood sizes (*k*).

The proposed EBCR approach has two main parameters, i.e. the neighbourhood size (number of neighbours whose ratings are used for rating prediction) and the parameter *a* in Eq (7), which parameterizes the size of the *neutral* area of the user taste modelling. The neighbourhood size is the same parameter as for basic memory-based CF approaches and the ones integrating with the Laplace smoothing (*LS*) or the *significance weighting* (*SW*). For each dataset, we considered 8 different neighbourhood sizes: {5, 10, 20, 40, 60, 80, 100, 200} for the two movie datasets and {5, 10, 15, 20, 25, 30, 35, 40} for the Jester joke dataset. We set the parameter *a* of the proposed approach at 0.5 after observing that it had a negligible impact on the final results. Specifically, to determine the value of the parameter *a* in Eq (7), experiments were conducted on the MovieLens-1M dataset with different *a* values for different neighbourhood sizes. As a result (Fig 1), we observed that the variation of the parameter *a* had a negligible impact on the final results and the value of *a* = 0.5 leads to slightly better results when the neighbourhood size is small.

Another parameter for memory-based collaborative filtering approaches is related to the way one builds the similarity matrix, i.e. user-based or item-based. For the *Jester* joke dataset, the experiments were conducted with item-based collaborative filtering in order to facilitate computations. As the joke dataset contains only 140 items comparing to 59.1K users, it is more flexible to adopt the item-based approach. In this way, the size of the similarity matrix (140 × 140) was much smaller than that built for the user-based approach (59.1K × 59.1K), which requires much more computer memory. For the movie datasets, the experiments were conducted using the user-based approach.

Regarding the specific parameters of the compared adjustment approaches, i.e. the significance weighting (*SW*) and the Laplace smoothing (*LS*), we followed recommendations provided by the authors of the corresponding works [13, 30]. Thus, the threshold *t* used in the *SW* approach Eq (6) was set to 50 and the pseudocount parameter *α* for the *LS* approach was set to 1 (which is equivalent to using a uniform prior in a Bayesian perspective).

Regarding the parameters and implementation of the model-based approaches, we followed the configurations provided in the corresponding original papers. To achieve this, we made use of existing open-source packages and implementations. Specifically, the *Baseline*, SVD, SVD++ models were implemented using the *Surprise* Python package [31], as was also the case for all considered memory-based approaches, including the proposed EBCR one. The package is dedicated to the development and evaluation of collaborative filtering algorithms within a homogeneous framework. For the NeuMF model, we followed an existing implementation [32] using the *PyTorch* framework.

All source code of the proposed EBCR method is provided at [33].

## Results

**Fig 2:**
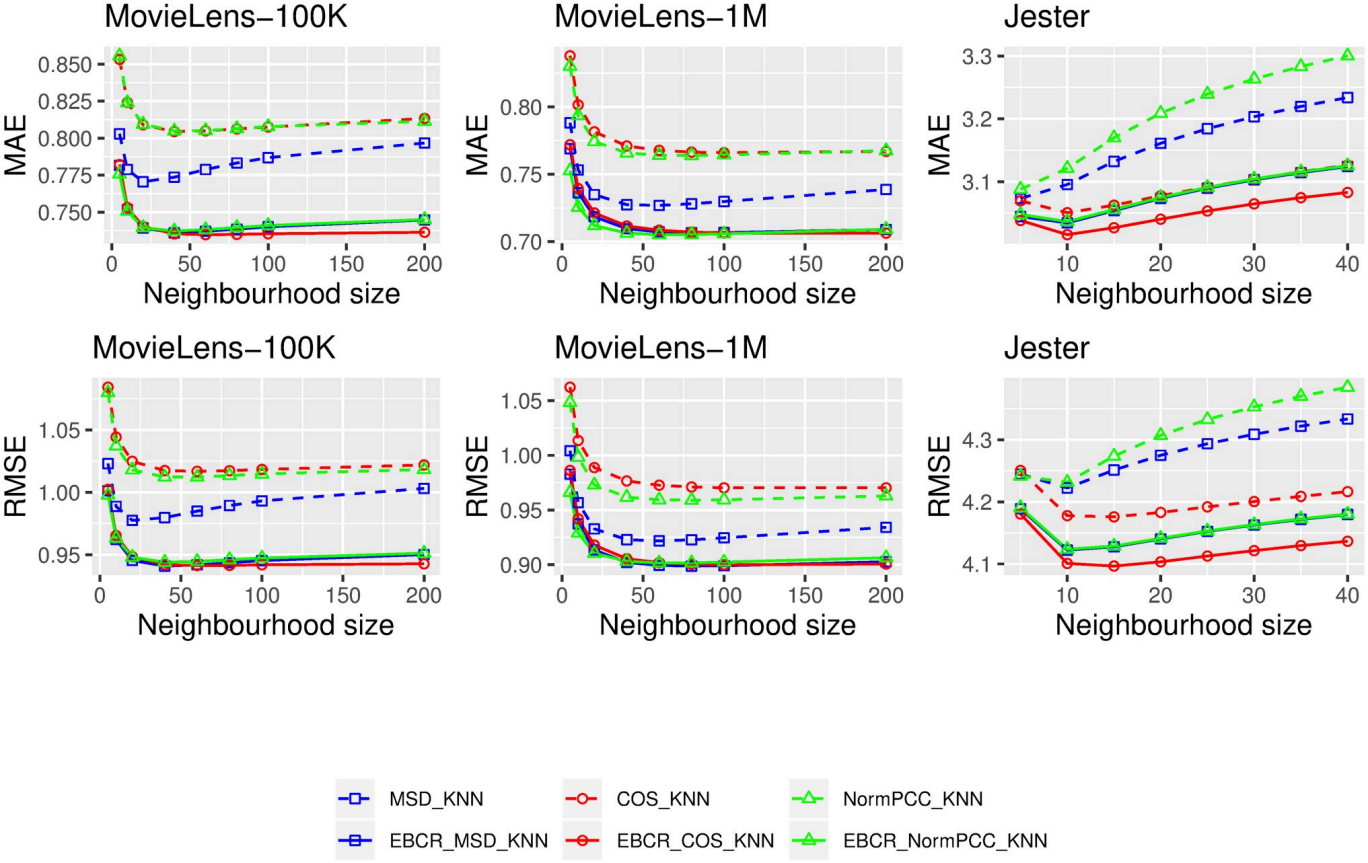
Comparisons of original similarity measures (i.e. dashed curves) with their variants integrating EBCR ratios (i.e. solid curves with the same colour) on three benchmark datasets: MovieLens-100K, MovieLens-1M and Jester.

Results of the three types of comparison are presented in this section. First, the results regarding the genericity of the proposed EBCR adjustment are shown in Fig 2. Each similarity measure in Fig 2 is associated with two curves of the same colour: the dashed ones for original measures (e.g. *MSD_KNN*, in blue with rectangles) and the solid ones for variants integrating the EBCR adjustment (e.g. *EBCR_MSD_KNN*). The solid lines are systematically below the dashed ones. For example, the EBCR adjustment led to a MAE/RMSE improvement of 7.78%/7.26% for the *COS_KNN* approach with a neighbourhood size equals to 100 for the *MovieLens-1M* dataset. The obtained results confirmed that the integration of the EBCR method is relevant for improving the overall performance of the tested collaborative filtering methods. They also confirmed the genericity of the contribution because the integration of EBCR improved the accuracy of rating predictions for all tested datasets and all similarity measures.

**Fig 3:**
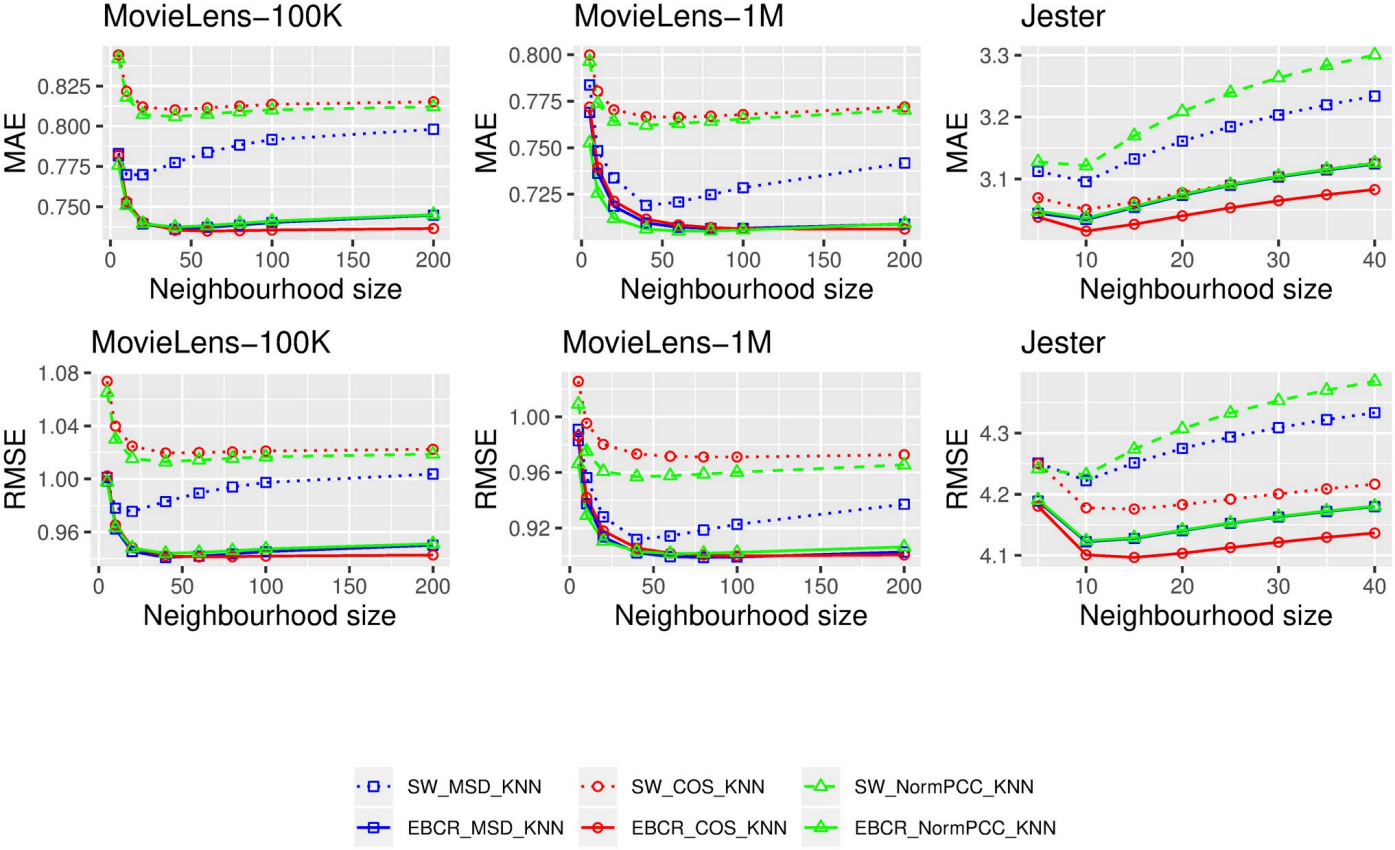
Comparisons of the *Significance Weighting (SW)* with the EBCR approach on three benchmark datasets: MovieLens-100K, MovieLens-1M and Jester.

Second, the results regarding the comparison of the proposed EBCR adjustment with the *SW* and *LS* adjustment approaches are respectively shown in Fig 3 and Table 2. As in Fig 2, in Fig 3 we show the obtained results by associating each similarity measure with two curves of the same colour: the dotted ones for similarity measures weighted by the *SW* factor and the solid ones for measures that integrate EBCR. Table 2 represents MAE and RMSE values of the *LS* and the EBCR approaches. As similar results were observed for the other neighbourhood sizes, in Table 2 we only illustrate results on these benchmark datasets with 4 different neighbourhood sizes. Meanwhile, all the results are provided at [33].

**Table 2:** Comparisons of the Laplace smoothing (*LS*) with the EBCR approach on three benchmark datasets: MovieLens-100K, MovieLens-1M and Jester.

Dataset	Similarity measure	Evaluation metric (LS, EBCR)	Neighbourhood size			
			5	10	20	40
MovieLens-100K	MSD	MAE	(0.790, **0.782**)	(0.758, **0.752**)	(0.743, **0.739**)	(0.738, **0.736**)
		RMSE	(1.010, **1.001**)	(0.969, **0.962**)	(0.949, **0.945**)	(0.943, **0.941**)
	COS	MAE	(0.788, **0.782**)	(0.757, **0.753**)	(0.743, **0.740**)	(0.737, **0.735**)
		RMSE	(1.009, **1.002**)	(0.970, **0.965**)	(0.951, **0.948**)	(0.944, **0.942**)
	NormPCC	MAE	(0.786, **0.776**)	(0.756, **0.751**)	(0.743, **0.740**)	(0.739, **0.737**)
		RMSE	(1.008, **0.998**)	(0.969, **0.963**)	(0.951, **0.948**)	(0.945, **0.944**)
MovieLens-1M	MSD	MAE	(0.782, **0.769**)	(0.747, **0.736**)	(0.727, **0.719**)	(0.717, **0.710**)
		RMSE	(0.997, **0.983**)	(0.949, **0.937**)	(0.923, **0.914**)	(0.909, **0.902**)
	COS	MAE	(0.781, **0.772**)	(0.747, **0.739**)	(0.727, **0.721**)	(0.716, **0.712**)
		RMSE	(0.997, **0.986**)	(0.950, **0.942**)	(0.924, **0.918**)	(0.909, **0.906**)
	NormPCC	MAE	(0.775, **0.753**)	(0.742, **0.726**)	(0.724, **0.712**)	(0.714, **0.706**)
		RMSE	(0.990, **0.966**)	(0.945, **0.929**)	(0.922, **0.911**)	(0.910, **0.903**)
Jester	MSD	MAE	(3.045, 3.045)	(3.035, 3.035)	(3.074, 3.074)	(3.125, **3.124**)
		RMSE	(4.189, 4.189)	(4.123, **4.122**)	(4.141, **4.140**)	(4.180, 4.180)
	COS	MAE	(3.039, **3.038**)	(3.017, **3.016**)	(3.041, **3.040**)	(3.083, 3.083)
		RMSE	(4.181, **4.180**)	(4.101, 4.101)	(4.104, **4.103**)	(4.137, 4.137)
	NormPCC	MAE	(3.049, **3.048**)	(3.037, 3.037)	(3.075, 3.075)	(3.126, 3.126)
		RMSE	(4.191, 4.191)	(4.124, 4.124)	(4.142, **4.141**)	(4.181, **4.180**)

The best value for each (LS, EBCR) comparison is shown in bold characters.

On the one hand, the results in Fig 3 illustrate that for all 72 tested conditions (3 datasets × 3 similarity measures × 8 neighbourhood sizes) the EBCR adjustment led to better MAE and RMSE values than those obtained with the *SW* approach. On the other hand, as shown in Table 2, slightly but systematically better results were obtained by the EBCR approach compared to the *LS* one. This last point shows that considering the whole sample taste distribution (as is the case for EBCR) was more relevant than the *ignorance* (i.e. the uniform prior as with the LS approach).

Third, the results concerning the comparison between the proposed EBCR adjustment and the model-based collaborative filtering approaches, including *Baseline*, SVD, SVD++ and NeuMF, are illustrated by Table 3. The datasets in Table 3 are listed by ascending order of density. The dataset density reflects the percentage of available ratings in the ratings matrix. This attribute is known to influence the relative performances of recommender system approaches [34, 35]. Model-based approaches in general perform better than memory-based ones on low density matrices. Conversely, memory-based methods tend to perform better on dense matrices. The results in Table 3 show that when the density of the dataset increases, the EBCR approach, which is a memory-based *CF* approach could outperform model-based approaches. For the *MovieLens-1M* dataset (density = 4.47%), the best method is SVD++ (MAE = 0.6729, RMSE = 0.8625) followed by SVD and NeuMF approaches. For the *MovieLens-100K* dataset, which is slightly denser (density = 6.30%), SVD++ is still the best method (MAE = 0.7214, RMSE = 0.9203), but EBCR comes next, ahead of other models in terms of MAE (0.7348). Finally, on the densest *Jester* dataset (density = 20.53%), the EBCR approach has the best results (MAE = 3.0158, RMSE = 4.1008), followed by NeuMF (MAE = 3.0375, RMSE = 4.1376) and clearly outperformed SVD (MAE = 3.3713, RMSE = 4.5004) and SVD++ (MAE = 3.6209, RMSE = 4.9042) models.

**Table 3:** Comparisons of EBCR vs. model-based collaborative filtering approaches.

	Dataset (density)					
Approach	MovieLens-1M (4.47%)		MovieLens-100k (6.30%)		Jester (20.53%)	
	MAE	RMSE	MAE	RMSE	MAE	RMSE
Baseline	0.7195	0.9088	0.7484	0.944	3.3982	4.3134
SVD	0.6863	0.8743	0.7376	0.9358	3.3713	4.5004
SVD++	**0.6729**	**0.8625**	**0.7214**	**0.9203**	3.6209	4.9042
NeuMF	0.6773	0.8765	0.7437	0.9363	3.0375	4.1376
EBCR	0.7052	0.9016	0.7348	0.9413	**3.0158**	**4.1008**

The best MAE and RMSE values for each dataset are shown in bold characters and the second ranking ones are underlined. For EBCR, the similarity measure (SM) and the neighbourhood size (NS) used for each dataset are as follows: SM = COS, NS = 60 for MovieLens-1M; SM = COS, NS = 60 for MovieLens-100k and SM = COS, NS = 10 for Jester.

Interestingly, the results presented in Table 3 suggest that conventional CF models e.g. SVD, SVD++ and EBCR could outperform the deep neural network (DNN)-based model (NeuMF) in terms of rating predictions. Although representative, the considered NeuMF model might not show the overall performance of DNN-based models. Notably, as many other DNN-based models compared in [10], the NeuMF model was designed to deal with implicit ratings and evaluated through *top-N* based metrics. As the evaluation protocol in this paper was based on explicit ratings (RMSE, MAE), the NeuMF model was adapted by using homologous loss functions to deal with the rating prediction task. However, works in [24, 36] have shown that DNN-based models clearly outperformed conventional models in the top-N recommendations scenario.

### A case study example

This subsection illustrates a real case study example extracted from the MovieLens-100K dataset to show the contribution of the EBCR method. Each of the compared approaches is asked to predict the rating value of the target user_903 (with 903 being the user ID) for the item_106 (with 106 being the item ID). The user’s actual rating (ground truth) for the item is 2. Table 4 shows the obtained results. For memory-based collaborative filtering methods, the neighbourhood size is fixed to 5 and the cosine measure is used to compute similarity between users. As shown in Table 4, the target user’s neighbourhood computed by the COS_KNN approach is completely different from the EBCR_COS_KNN one. Specifically, Table 4 shows that for the COS_KNN approach, all of the numbers of items co-rated by the target user and his/her neighbours are small, e.g. the neighbours user_36 and user_33 co-rated 2 items with the target user. While in the EBCR_COS_KNN case, users included in the target user’s neighbourhood have more items co-rated with the target user, e.g. the neighbours user_556 and user_8 co-rated respectively 13 and 18 items with the target user. The above observations illustrate how those users who co-rated few items with the target user are excluded from the neighbourhood during the EBCR adjustment, even though they shared the same ratings for their few co-rated items. Regarding the predicted rating, Table 4 shows that the EBCR_COS_KNN leads to more accurate rating prediction compared to the COS_KNN approach, i.e. 2.306 vs. 3.195 (while the ground truth rating value is 2). Moreover, for the considered case study example, the results show that the EBCR approach achieves the best prediction compared to all other methods.

**Table 4:** A case study example extracted from the MovieLens-100K dataset.

Case study: predict the rating of user_903 (target user) for item_106 (the user’s actual rating is: 2)		
Approach	Neighbours (# of co-rated items with the target user)	Predicted rating
COS_KNN (k = 5)	user_36 (2); user_33 (2); user_240 (3); user_61(1); user_173 (2)	3.195
EBCR_COS_KNN (k = 5)	user_556 (13); user_8 (18); user_898 (4); user_563 (7); user_609 (4)	**2.306**
Baseline		3.122
SVD		2.925
SVD++		2.780
NeuMF		2.934

The best prediction approach is bolded and the second ranking one is underlined.

## Conclusion & future work

In this article a new method is proposed to refine the similarity estimation between users in the memory-based collaborative filtering context. The proposed concordance ratio between two users represents the concordance of their ratings. It takes the potential disparity of their rating systems into account. In addition, we proposed to adjust each of these ratios using the Empirical Bayes prior, which takes into account the distribution of all concordance ratios within the training set. Moreover, this Bayesian adjustment accounts for the number of items that two users have co-rated, a factor that is typically overlooked by existing similarity measures. The assessment of our approach on benchmark datasets confirmed that it systematically improved the rating prediction accuracy.

The proposed approach has two major advantages: simplicity and genericity. Indeed, the adjusted ratio, denoted EBCR, as a smoothing of widespread similarity measures, is easy to integrate and does not increase calculation time. In addition, the contribution of the EBCR approach seems generic as it improves the quality of the neighbourhood-based collaborative filtering for all tested conventional similarity measures, regardless of the neighbourhood size considered.

The comparison results concerning the proposed method vs. model-based CF methods also highlighted the relevance of the EBCR adjustment especially in the context of dense datasets, e.g. the proposed approach could outperform neural network-based methods on the Jester dataset. Nevertheless, as discussed at the end of the Results section, different recommendation scenarios may lead to one model appearing more appropriate than another. Specifically, when dealing with rating predictions (*single items*), conventional CF models would be more appropriate than models based on deep neural networks. The latter could be a better choice within a top-N recommendations context (*list of items*). Note that in some real-world applications, rating predictions on single items could be more useful such as recommendations for a group of users [37] where it is difficult to provide top-N recommendations by treating the user group as a whole.

Another example [38] could be to show users the predicted rating of each item, thus helping them make decisions. Moreover, recommendations generated by neighbourhood-based CF are easier to explain than *black box* models based on latent factors. For example, the explanation pattern could be *“users who bought item i like you, also bought item j*”. Nevertheless, classic neighbourhood-based approaches do not distinguish whether or not the recommendations are based on trustworthy neighbours. To this end, the proposed EBCR method could also be used to enhance users’ trust in their recommendations because the selection of items recommended is based on neighbours who have co-rated a considerable number of items and are therefore more reliable.

Lastly, the proposed concordance ratios are based on the cardinality of co-rated items by user pairs, while ignoring content/knowledge information about items, e.g. starring, director etc. for movies. Each of these ratios is thus adjusted using the same parameters of the prior Beta distribution, i.e. *α*_0_ and *β*_0_. Future works might investigate how the ratio adjustment could be personalized via the integration of item knowledge.
